# A Case of Left Retroexternal Iliac Artery Megaureter Associated with Additional Renal and Vascular Congenital Anomalies

**DOI:** 10.1155/2020/8946435

**Published:** 2020-07-24

**Authors:** Amin A. Karadaghy, Matthew T. Bell, Daniel T. Daly, Yun Tan

**Affiliations:** Center for Anatomical Science and Education, Department of Surgery, Saint Louis University School of Medicine, Saint Louis, MO 63104, USA

## Abstract

*Introduction*. A number of rare anatomical anomalies, including retroexternal iliac ureter, extrarenal calyces (ERCs), and vascular anomalies, were observed in a 96-year-old female cadaver during a routine dissection. *Description*. A markedly dilated left extrarenal pelvis (ERP) with a diameter of 3.15 cm was noticed. Three major calyces were found outside of the normal-sized left kidney. The abdominal aorta (AA), instead of normal bifurcation, branched to the right common, left external, and left internal iliac arteries. The median sacral artery was a direct branch from the right common iliac artery. No hydronephrosis was observed on the affected side, and no urinary tract anomalies were observed on the right side. *Significance*. The retroiliac megaureter is a rare congenital anomaly, with fewer than 25 cases reported to date. Additionally, the ERCs are amongst the rarest anomalies of the renal collecting system. Further, the current case is one of few reported cases where the particular branching pattern of the AA was observed. The combination of such anatomical anomalies is rare, and the relationship between them is unclear. Common clinical manifestations of retroiliac ureters are the results of ureteric obstruction, hydronephrosis, and secondary infection. Precise knowledge of anomalies of the kidney and urinary tract can help radiologists and surgeons make a definitive diagnosis and prevent inadvertent injury during surgery.

## 1. Introduction

Congenital abnormalities of the kidney and urinary tract (CAKUT) encapsulates a wide range of structural abnormalities that arise during the development of the kidneys and urinary tract. CAKUT represents 20% of all birth defects and is a major cause of pediatric end-stage renal disease [1]. Known risk factors for CAKUT include intrauterine exposures, such as exposure to ACE inhibitors, and family history [2].

One well-described form of CAKUT is megaureter, defined simply as a ureter with a larger than normal diameter with or without involvement of the renal pelvis. A normal ureter is ~25-30 cm in length from the renal pelvis to the bladder and ~3-5 mm in diameter along its length. Megaureters are divided into four categories: obstructive, refluxing, refluxing with obstruction, and nonrefluxing/nonobstructing [3]. These four categories are further subdivided into two subcategories: primary (vesicoureteral junction dysfunction) or secondary (bladder obstruction) [4]. The decision to begin treatment is multifactorial and depends on symptomology, severity, and underlying conditions [5].

Secondary obstructive megaureter is most commonly due to obstruction at the level of the bladder by posterior urethral valves [6, 7]. This occurs most commonly on the left side and in males due to a remnant of embryological membranes [5, 8].

Much less commonly, secondary obstructive megaureter can be due to a retroiliac ureter wherein the ureter passes deep to the iliac artery. There have been a very small number (24) of documented retroiliac ureter cases reviewed to date [9]. Obstructive megaureter is associated with several anatomic variations including retro-common iliac artery (CIA) with lack of internal iliac artery (IIA), retro-IIA, and circumiliac ureter [7, 10, 11].

In the current report, we describe a novel case of left retro-external iliac artery (EIA) megaureter. This case includes other renal and vascular anomalies, specifically the absence of a left CIA, the presence of ERC, and a unique AA branching pattern. Because this case was found postmortem, it permitted detailed exploration and measurement.

This case is of interest for its rarity and clinical implications. The importance of understanding vascular anatomical variations is highlighted in a case of development of the left lower limb ischemia following a routine nephrectomy due to an aberrant EIA originating from the left renal artery [12]. This case emphasizes the importance of how knowledge of anatomical variations can help clinicians exercise caution in a clinical setting.

## 2. Case Presentation

A 96-year-old female body was received through the Saint Louis University Gift of Body Program of the Center for Anatomical Science and Education (CASE) with the signed informed consent from the donor. The CASE gift body program abides by all rules set forth by the Uniform Anatomical Gift Act (UAGA). Information obtained through the CASE program did not specify a cause of death but did mention a history of Alzheimer's disease, COPD, and atrial fibrillation in this donor. During dissection in 2018, it was observed that the donor was status post hysterectomy and status post ventral hernia repair with mesh.

**Figure 1:**
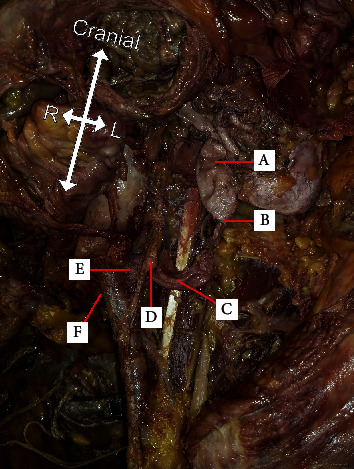
Dissected path of left ureter from the kidney to the iliac arteries. (A) The beginning of the renal pelvis (diameter: 3.15 cm), (B) the end of the renal pelvis (diameter: 0.88 cm), (C) passing over psoas minor (diameter: 1.70 cm), (D) passing under ovarian artery/vein, (E) passing under external iliac artery (diameter: 1.65 cm), and (F) emerging medial to external iliac artery (diameter: 0.45 cm).

During routine dissection, a markedly dilated left ureter was noticed and further explored (Figure 1). The left renal pelvis was located outside of the kidney sitting medially, inferiorly, and slightly posteriorly from the kidney. The path of the ureter curved slightly posteriorly and medially before turning laterally. The ureter then turned sharply medially and anteriorly before continuing over the psoas major muscle. The ureter was most dilated as it passed anteriorly to the left psoas minor muscle, where its diameter measured at 1.70 cm. The ureter then passed posterior to the EIA and anterior to the IIA, about 0.50 cm away from the bifurcation of the AA.

**Figure 2:**
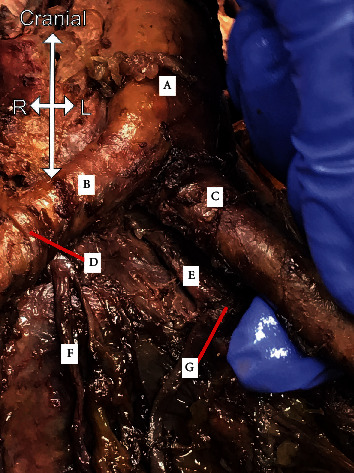
Terminal branches of the abdominal aorta and paths of ureters. (A) Abdominal aorta, (B) right common iliac artery, (C) left external iliac artery, (D) right ureter passes over common iliac artery, (E) let internal iliac artery, (F) median sacral artery branches off the right common iliac artery, and (G) the left ureter passes between the left internal iliac artery and external iliac artery.

**Figure 3:**
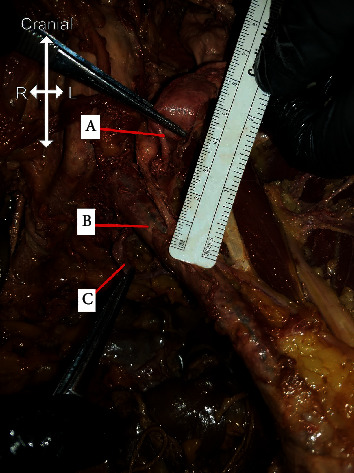
Ureter passes beneath the left external iliac artery and emerges distally with a normal diameter. (A) Proximal, preobstruction end of ureter, (B) the point of obstruction under the left external iliac artery, and (C) distal, postobstruction end of the ureter.

Upon further dissection, it was found that what was initially identified as the left CIA was in fact the left EIA. The donor had three terminal arterial branches off the AA: the right CIA, left IIA, and left EIA. The left IIA branched off the posterior side of the AA. As a result, the median sacral artery was shifted and was observed branching off the right CIA (Figure 2). After passing beneath the left EIA, the left ureter returned to a normal size (0.45 cm) and position following a normal path to the bladder (Figure 3).

**Figure 4:**
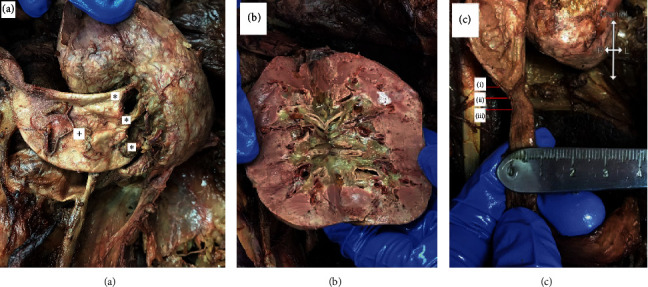
(a) A posterior view of the affected (left) kidney showing three major calyces (asterisks) joining outside of the kidney and a dilated renal pelvis (plus sign). (b) A view inside the affected (left) kidney showing normal-sized minor calyces and pyramids without evidence of hydronephrosis. (c) Also notable was a slight, yet still noticeable, stenosis of the ureteropelvic junction: (i) pre-ureteropelvic junction (diameter: 0.88 cm), (ii) ureteropelvic junction (diameter: 0.50 cm), (iii) post-ureteropelvic junction (diameter 0.75 cm).

The three major calyces of the left kidney were found to join together outside of the kidney (Figure 4(a)). All arteries and veins of the left kidney were normal, and no evidence of hydronephrosis was observed (Figure 4(b)). Constriction of the ureteropelvic junction (UPJ) was noted, though this slight constriction did not result in any gross renal pathology (Figure 4(c)).

Finally, the subject exhibited extensive surgical sutures, likely from the hysterectomy, along the lateral paravertebral muscles as well as near the left IIA, inferolateral to the ureter.

## 3. Discussion

The only review found in the literature on retroiliac ureter described 24 cases, many of which were associated with various genitourinary anomalies including those of ectopic vas deferens, horseshoe kidney, and imperforate anus [13]. The current case appears to be the first reported with the constellation of various anomalies including a left retro-EIA ureter with ERC and absent left CIA with a unique AA branching pattern. The importance of the present report is several fold.

*First*, this case highlights yet another form of secondary obstructive megaureter wherein the aberrant ureter is obstructed between the IIA and EIA and thus may be called retro-EIA megaureter. Similar cases of retro-IIA, retro-CIA, and circumiliac artery ureter have been previously described [7, 10, 11]. More commonly, secondary obstructive megaureter is caused by aneurysm of the iliac arteries, arteriovenous malformation, and persistent umbilical artery [14–20]. These common causes of obstructive megaureter were not observed in the cadaveric body under study, rather the obstruction was due to the ureter coursing posterior to the EIA.

*Second*, the observed branching pattern of the AA seems to be very unique. As noted above, the current cadaveric body lacks a left CIA and the left IIA was observed branching directly off the posterior side of the AA with the left EIA arising directly from the lateral side of the AA. Generally, cases of vascular anomalies in the iliac and femoral vessels are rare [21]. One study reported the case of the unilateral lack of iliac arteries, with a network of collateral arteries supplying the normal vascular territory [21]. Similar cases include absence of CIA and IIA with an aberrant EIA arising from the renal artery, absence of right CIA with aberrant left IIA, and absence of bilateral CIA and left IIA with prominent collateral vasculature [12, 22, 23]. A very similar iliac artery branching pattern to this case has been described but included absent bilateral, rather than unilateral, CIAs [24]. Variations in terminal branches of iliac and femoral arteries have been described more thoroughly, with several classification systems used to categorize variations in iliac artery branches [25].

*Third*, this case is notable for its associated urinary tract anomalies, namely dilated ERP, ERC, and UPJ stenosis, all present with a conspicuous absence of hydronephrosis. ERP is an anatomical variant that appears just outside the renal sinus and can be confused with hydronephrosis, especially on renal ultrasound, but can be distinguished by its lack of dilated calyces, parenchymal thinning, hydroureter, or enlarged kidney [26]. ERP is estimated to be seen in up to 10% of the population and is asymptomatic in most cases. Extrarenal calyces (ERCs) are among the rarest anomalies of the renal collecting system, with only 20 cases reported to date [19]. The ERC association with UPJ obstruction is described only rarely in the literature [27]. The etiology of ERC is unclear and is most commonly associated with ectopic kidney, which is usually the result of variation in degrees of embryological kidney nonrotation [19]. The clinical presentation of ERC includes flank pain and recurrent UTIs usually as a result of stasis, calculi, and infection [19, 28]. It has been diagnosed incidentally during cadaveric dissection or investigation of hydronephrosis [19, 27]. As described by one paper, treatment is not always necessary but includes either a classic pyeloplasty, resection of the pelvis, and an ureterocalicostomy with fusion of the calyces, or a single-stage nephroureterectomy [27]. Interestingly, no other genitourinary anomalies were present in this body. The bladder and urethra were examined and no abnormalities regarding size or positioning were noted.

*Fourth*, this case is notable because it provides important information regarding embryologic aspects. The ureter originates from the ureteric bud, an evagination of the mesonephric (Wolffian) duct that forms during the fourth or fifth week of gestation. Through repeated rounds of elongation and branching, the buds eventually mature into the bilateral ureters [29]. Starting in the sixth or seventh week of gestation, the developing kidney migrates rostrally, passing anterior to the iliac vessels which develop as branches off the umbilical artery. Abnormalities in this migration are generally considered to be a cause of secondary obstructive megaureter arising from an aberrant ureter [7]. Therefore, it would be reasonable to suggest that the aortic abnormalities represent the primary aberration and not the ureteric migration. For the same reason, a similar condition previously called retrocaval ureter was redefined as the preureteral vena cava [30]. The genetic programming underlying ureteric bud positioning and migration has been implicated in the development of retroiliac ureter and extensively studied [29, 31–34].

*Fifth*, this case is also important because it could modify the respective prenatal screening to make it more accurate and informative. CAKUT is a major cause of chronic kidney disease in children, often resulting in end-stage renal disease thereby requiring a kidney transplant [35]. CAKUT and its associated abnormalities are often detected during prenatal ultrasound examination, which is recommended by the American College of Obstetricians and Gynecologists to be performed at least once during pregnancy. One retrospective study of congenital malformations showed that 73% of cases of congenital hydronephrosis were diagnosed prenatally [8]. In countries where prenatal ultrasound examination is rare, postnatal ultrasound in children with UTI, or those at higher risk, could also detect congenital abnormalities [36, 37]. According to other publications, the incidence of prenatally diagnosed megaureter is not currently known and may be due to the fact that detection of a dilated ureter by ultrasound prenatally is a difficult endeavor [8].

Finally, this case has a high clinical value. Indeed, surgical treatment of retroiliac ureter is most commonly done by transposition of the ureter around the obstruction. Other documented treatments include iliac artery diversion, nephroureterectomy, and transureteroureterostomy [13]. Knowledge of the anatomical variants was highlighted by a report where an anomalous EIA arising from the renal artery was unexpectedly compromised during a nephrectomy [12].

## 4. Conclusion

This postmortem case of a novel retroexternal iliac artery megaureter with absent left common iliac artery and presence of extrarenal calyces is of interest for both its rarity and clinical significance. The embryological etiology of this particular genitourinary and vascular variant is unclear at this time. However, this variant does pose clinical significance for the medical and surgical community. Common clinical manifestations of retroiliac ureters are usually the result of obstruction and secondary infection though, to our knowledge, this patient was not experiencing any of those. Furthermore, identifying the location and orientation of anatomical details of the abdominal aorta and iliac vasculature as well as ureteral anomalies is essential for performing various pelvic surgeries ranging from uterine fibroid removal to hernia repair. Failure to consider these potential variations can lead to preventable morbidity and mortality during surgery.
